# Water and seawater splitting with MgB_2_ plasmonic metal-based photocatalyst

**DOI:** 10.1038/s41598-024-82494-5

**Published:** 2025-01-07

**Authors:** Vasyl G. Kravets, Alexander N. Grigorenko

**Affiliations:** Department of Physics and Astronomy, the University of Manchester, Manchester, M13 9PL UK

**Keywords:** Nanoscience and technology, Optics and photonics, Physics

## Abstract

Plasmonic nanostructures can help to drive chemical photocatalytic reactions powered by sunlight. These reactions involve excitation of plasmon resonances and subsequent charge transfer to molecular orbitals under study. Here we engineered photoactive plasmonic nanostructures with enhanced photocatalytic performance using non-noble metallic MgB_2_ high-temperature superconductor which represents a new family of photocatalysts. Ellipsometric study of fabricated MgB_2_ nanostructures demonstrates that this covalent binary metal with layered graphite-like structure could effectively absorb visible and infrared light by excitation of multi-wavelengths surface plasmon resonances. We show that a MgB_2_ plasmonic metal-based photocatalyst exhibit fundamentally different behaviour compared to that of a semiconductor photocatalyst and provides several advantages in photovoltaics applications. Excitation of localised surface plasmon resonances in MgB_2_ nanostructures allows one to overcome the limiting factors of photocatalytic efficiency observed in semiconductors with a wide energy bandgap due to the usage of a broader spectrum range of solar radiation for water splitting catalytic reactions conditioned by enhanced local electromagnetic fields of localised plasmons. Excitation of localised surface plasmon resonances induced by absorption of light in MgB_2_ nanosheets could help to achieve near full-solar spectrum harvesting in this photocatalytic system. We demonstrate a conversion efficiency of ~ 5% at bias voltage of *V*_*bias*_ = 0.3 V for magnesium diboride working as a catalyst for the case of plasmon-photoinduced seawater splitting. Our work could result in inexpensive and stable photocatalysts that can be produced in large quantities using a mechanical rolling mill procedure.

**Supplementary Information:**

The online version contains supplementary material available at 10.1038/s41598-024-82494-5.

## Introduction

The hydrogen economy is a sunrise industry considered by many as the ultimate solution to power the future society. As a potential technology for hydrogen production, photocatalytic water splitting is the most promising. Sunlight is a clean and inexhaustible energy gift from nature. The solar radiation reaching the Earth’s surface comprises nearly 100,000 TW power, which is much more than that of the current global power consumption (~ 16.3 TW)^1–4^. Practically, the harvesting of solar energy takes place from only a 0.07% Earth’s land surface area of the total amount solar. It was theoretically predicted that converting such amount solar energy into usable form of energy (hydrogen and electricity) with 10% efficiency could cater the global energy demand^3–6^. Unfortunately, currently the energy conversion efficiency of photocatalytic water splitting is still too low for large-scale applications (the H_2_-production activities do not achieve a solar-to-hydrogen (STH) efficiency of over 10%) and a widespread usage of hydrogen as green energy. The present situation in green H_2_-production demands further developments in nanomaterial engineering to potentially solve the problems of low photoanode effectivity^7,8^.

Deeper understanding of natural photosynthesis can bring the state-of-the-art modern approaches for designing photocatalytic systems. Usually, for photosynthesis, plants use the water from soil that contains lot of different impurities. On the other hand, in artificial photosynthesis, the direct production of green hydrogen on a large scale using seawater electrolysis is a long-sought dream of researchers^9,10^. There are only a handful of studies that demonstrated production of H_2_ from photocatalytic seawater splitting with large current densities at low overpotentials that can meet the industrial requirements, without deactivating the catalysts by the ions of sodium and chlorine^11,12^. A variety of photocatalysts for seawater splitting has been discussed: nitrides, transition metal hydroxides, sulphides and phosphide, rare earth metal nanocomposites^11,12^. A usage of seawater splitting for industrial production of H_2_ is very important task because the oceans represent 96.5% of the total water reserves of the planet that can provide an almost unlimited resource. It is interesting to note that the composition of seawater varies from region to region with the average overall salt concentration of all ions ranges at about 3.5 wt % gives pH ∼ 8^9,10^. At present, the best strategy to produce H_2_ from seawater is to split it into hydrogen and oxygen using electricity that comes from renewable energy sources, for example, photovoltaic cells. However, it would be much easier and cheaper if sunlight could be used directly to split seawater^13,14^. This requires new materials and novel mechanisms of photocatalytic seawater splitting. Moreover, to enhance efficiency of photo-water splitting significantly, it is necessary to develop materials that would absorb photons over the wide range of solar spectrum capable of water splitting (SSCWS). To achieve this, one can use metallic nanostructures with different sizes and shapes that would absorb photons over SSCWS due to excitation of the localised surface plasmon resonances (LSPRs) at multi-wavelengths.

Noble metal nanoparticles (NPs) made of Ag, Au, Cu, and Al are receiving increasing attention as photocatalysts, primarily due to the large absorption cross-sections in the visible light provided by their plasmon resonances. Under resonant excitation, the optical cross-sections of plasmonic nanostructures can be ~ 10 times higher than their geometric cross-sections. As a result, large amount of light energy is localised near the surface of plasmonic nanostructures in the form of intense local electromagnetic fields^15–22^. Thus, LSPR enhances photocatalysts’ performance through several mechanisms, which include plasmon-induced indirect hot-carrier transfer, direct charge transfer and intramolecular charge transfer, near-field effects and light scattering trapping^16,18–20,22^.

Initially, plasmonic photocatalysts were produced by combining plasmonic metal nanostructures with wide bandgap semiconductors (for example, TiO_2_) where noble metals NPs functioned as a photosensitizer to enhance the surface reaction on semiconductors^20,21^. It was shown that a dense array of aligned gold nanorods capped with TiO_2_ could produce 20 times higher efficiency for water splitting as compared to just TiO_2_ efficiency^23^. Linic and et al. were first to report direct molecular oxygen activation and ethylene epoxidation on plasmonic Ag nanoparticles^18,24^ which triggered an acceleration in the development of plasmonic photocatalysts^19–21^. The idea of plasmonic photo-catalysis is connected to the concentration of solar light near metal NPs by LSPR excitation and then subsequent direct electronic excitations of a material attached to the NPs^18,24–27^. The direct electron transfer pathway is expected to have higher transfer efficiency and lower energy losses in comparison to indirect ones^21^. Note that the direct interaction between plasmonic metal and its surface molecules was also confirmed by Halas’ group^16,28^. They demonstrated that hot electrons (high energy electrons) induced by LSPR excitations in Au NPs can be direct transferred into a Feshbach resonance of an H_2_ molecule adsorbed on its surface, causing its dissociation^16,28^. In review^29^ was discussed the plasmon excitations in metallic nanostructures based on noble metals as well as non-traditional plasmonic materials resulted in generation hot carriers for photocatalysis and light harvesting processes. Thomann and et al. demonstrated that plasmon-induced photochemistry to streamline chemical reactions can be enhanced by multilayer interference effects which promotes to high concentrate sunlight close to the electrode/liquid interface^30^. Note that the plasmonic nanostructures with strong optically resonant properties can behave as nanoscale optical antennas for solar light-harvesting and have shown extraordinary promise as light-driven catalysts^31^.

There are several reasons why metallic catalysts with plasmonic response could be best suited for direct water splitting by sunlight. In contrast to semiconductors, which generally exhibit poor chemical and catalytic activity due to the lack of the electron density at the Fermi level, plasmonic NPs possess significant electron density at the Fermi level, and strongly absorb ultraviolet (UV), visible (VIS) and near-infrared (near-IR) light^18^. The amount of light absorbed by a plasmonic material (the molar extinction coefficient of metal NPs) is in the range of 10^8^–10^10^ M^− 1^ cm^− 1^, which is approximately 10^4^–10^6^ times higher than most of the light absorbing species known^18,32^. This essentially means that plasmonic nanostructures can generate large number of high-energy electron–hole (e^−^ - h^+^) pairs upon irradiation with visible light (≈ 10^16^ cm^− 2^). Moreover, some plasmonic nanostructures can effectively utilise photons across the near-IR region wavelengths (750–2000 nm) which concentrates the approximately 50% of the solar energy spectra^1–3^. Unfortunately, mechanisms of the charge injection from excited plasmonic NPs to catalytic molecules are not well understood.

Here we report the room temperature water and seawater splitting on magnesium diboride (MgB_2_) nanostructures using VIS and near-IR light. We show that inexpensive MgB_2_ nanostructures provide efficient plasmonic water splitting and could be a viable alternative to noble metal nanoplasmonics. (It is worth noting that noble-like plasmonic metals, such as Cu, Ag, and Au typically exhibit low intrinsic activities with surface-adsorbed molecules due to their fully filled *d* bands^24,32^). An introduction of nanostructures based on non-noble metals with high intrinsic direct water splitting activities could present a promising strategy to harvest the solar energy efficiently. In MgB_2_ nanostructures (unlike in Au, Ag based) the LSPRs are strongly coupled with interband excitations due to a spectral overlap of these processes.

In our previous works we have shown that at the surface properties of MgB_2_ nanostructures can be actively involve into the process of water splitting in photoelectochemical cells (PECs)^33,34^. In this study, we demonstrate that interband damping of LSPRs is also an important channel for the formation of e^–^–h^+^ pairs. These e^–^–h + pairs are separated over two bands (namely, between the *π* and *σ* bands) have a longer lifetime and are thereby expected to harvest sun energy more efficiently and contribute to the generation of H_2_ together with direct electron injection mechanism of hot electrons generated via LSPRs. We also demonstrate that, in sharp contrast to semiconductor photocatalysts, photocatalytic efficiency on plasmonic-like MgB_2_ nanostructures increases for seawater splitting. More importantly, we suggest and test a new method of fabrication of plasmonic nanostructures with large area on flexible substrates for seawater splitting using mechanical rolling mill procedure which open up avenues to implement design that enable much-improved effectivity and costless of hydrogen production. This method provides stable and dense packed nanostructures.

## Experiments and results

### Studied materials

**Fig. 1:**
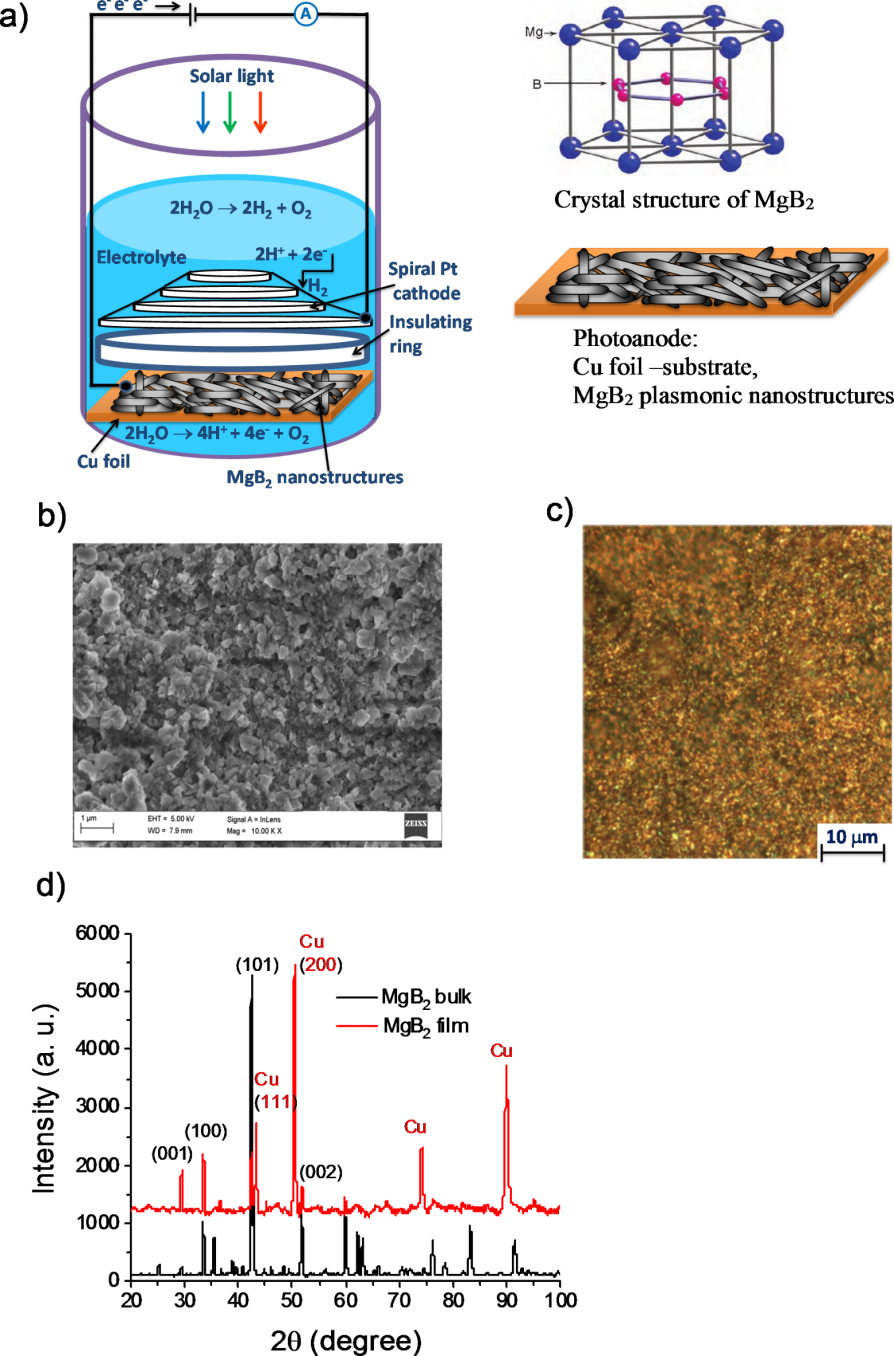
Magnesium diboride nanostructures as a model system for plasmonics photocatalys. **(a)** Schematic design of the integrated photoelectrochemical cell using MgB_2_ nanostructures as the photoanode and Pt cathode in different electrolytes. Right panels (a) layered structure of electrodes and hexagonal structure of MgB_2_ consisting of honeycomb B (red) layers with close-packed Mg (blue) layers between them. (**b**) SEM image showing the assembly of MgB_2_ nanostructured layers. (**c**) Optical image showing the assembly of MgB_2_ nanostructured layers which exhibit different colors due to excitation of different localised surface plasmon resonances. **(d)** X-ray diffraction pattern of the MgB_2_ nanostructured layer compared with XRD of bulk MgB_2_.

In this study, we focussed on MgB_2_, a binary compound composed of hexagonal boron sheets alternating with Mg cations, as the parent material in the top-down approach for borophene or borophene-related 2D sheets. Metal diborides MB_2_ typically exhibit a layered honeycomb structure with electron delocalisation within the M − M sublattice and strong covalence in the B − B sublattice, producing stable B − B bonds. It is well known that van der Waals (vdW) interactions between two atomic layers (Mg and B) play an important and crucial role in 2D materials to improve the electrochemical activity^6–8^. It is worth noting that diborides (like MgB_2_) could hold a promise for photoelectrochemical seawater splitting due to its stability, low-cost, abundance, appropriate bandgap and direct electrons injection into chemical reaction through the decay of plasmons. Figure 1a shows the schematics of the PEC used in our experiments.

### Sample fabrication

The layered structure of metal diborides with their graphene-like boron sheets suggests the possibility of exfoliation into thin nanosheets down to a monolayer^35^. Here we have developed new method of producing metal diborides as quasi-two dimensional (2D) nanosheets using a mechanical rolling mill procedure (Pepetools TM 90 mm Flat Rolling Mill). In this method, diboride powder can be used directly from commercial sources without any further purification. This powder was purchased from Sigma Aldrich with a stated purity of over 99%. Cu foils with thickness of less than 100 μm were used as the substrates. Before pressing, the squared surface of the Cu foil of size 20 × 20 mm was covered with powder totalling a mass of around 30 mg. Another Cu foil sheet was placed on the top of the powder, encasing it between two layers of Cu. To ensure the samples become very thin, they were ran through the rollers multiple times with increasing the force. Due to the fabrication process, the samples were stretched by up to 2 times in surface. This cheap, low-cost and quick method allows producing stable and dense samples that can be used in seawater splitting for a very long time. The produced nanostructures have a distribution of lateral dimensions from tens of nanometers up to several micrometers (0.1–5 μm) and a distribution of thicknesses from as low as few up to tens of nanometers (1–30 nm), which were measured by high resolution optical and scanning electron microscopies (SEM), Fig. 1b, c. The presence of LSPRs nature of fabricated MgB_2_ nanostructures is confirmed by multiple colours in the optical image, Fig. 1c. One can see that different areas of the sample changes colour from blue to red. In addition, one can see black spots corresponding to total light absorption. The multitude of colours can be explained by a variation of reflectivity characteristics due to excitation of multi-wavelengths LSPRs around the sharp edges of the nanosheets (Fig. 1). The structural properties of the plasmonic MgB_2_ photo-anode were determined by X-ray diffraction (XRD) technique using CuKα radiation (*λ* = 0.15418 nm) in the range of angles 2*θ* from 20° to 100°. Diffraction peaks were observed in the XRD pattern of the fabricated MgB_2_ sample. These peaks are associated with the MgB_2_ crystalline phases^36^ (Fig. 1d): 2*θ*≈29.6^o^ (001), 33.5^o^ (100), 42.4^o^ (101), 51.8^o^ (002), 59.9^o^ (110) and were also observed for the MgB_2_ sputtering target (bulk sample, purchased from American Elements). A few weak peaks at larger diffraction angles 2*θ* probably originate from the stacking periodicity of MgB_2_ sheets. Additional peaks for MgB_2_ nanostructured film on Cu substrate could correspond to the Cu crystalline phases^37^.

The resistance of the fabricated samples was determined using a digital multimeter. We found that resistance of the nanostructured photo-anodes (30–50 Ω, depending on thickness of flakes) was much larger as compared to that of thick MgB_2_ (< 1 Ω) measured on a square sample with a side of 10 mm. The initial contact voltage between plasmonic MgB_2_ photo-anode and Pt photo-cathode embedded in electrolyte in the dark condition was found to be *V*_*init*_∼ -0.7 eV in 0.5 M NaCl.

### Photocatalytic tests

**Fig. 2:**
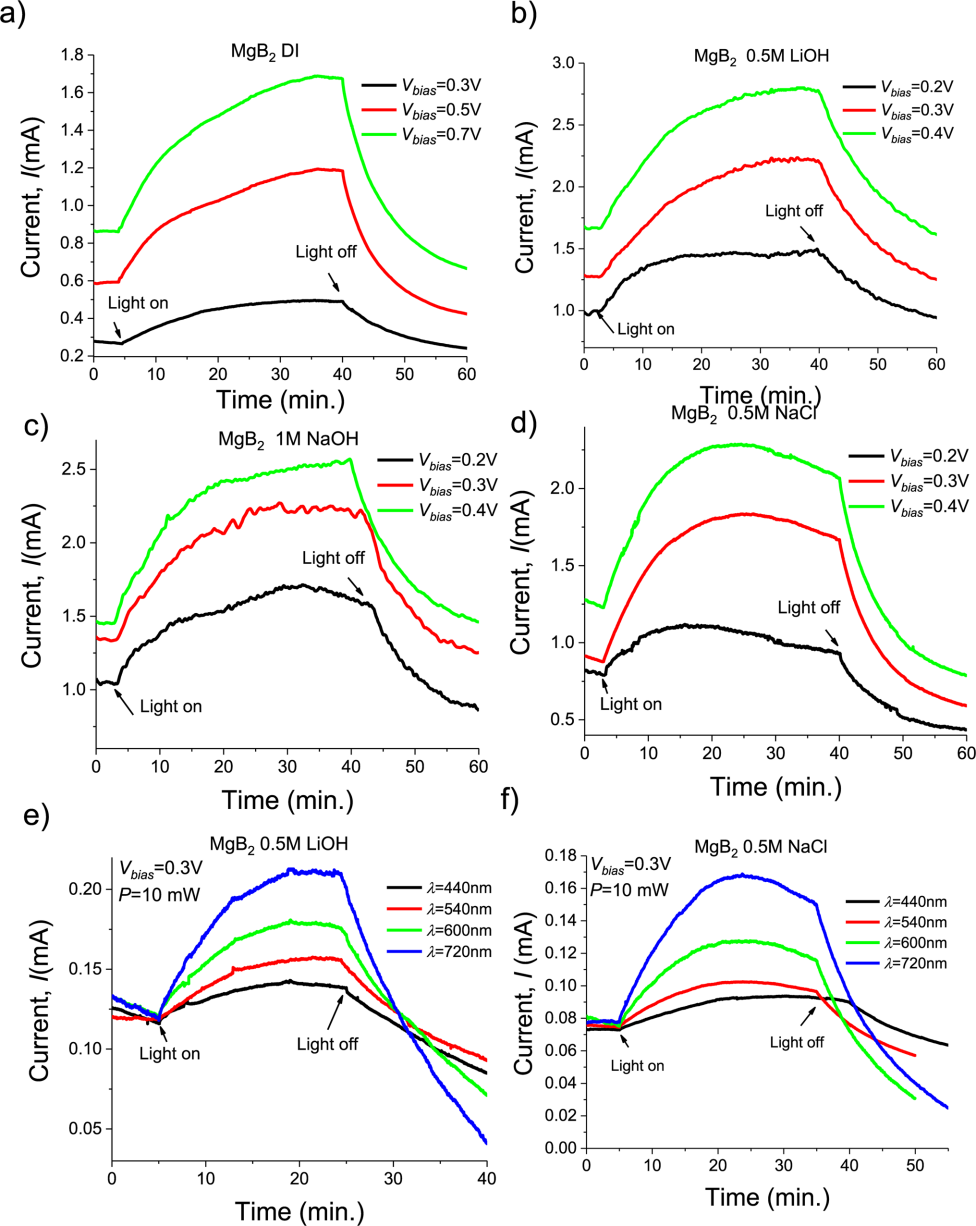
Solar water splitting. Photocurrent as a function of time and external voltage, *V*_*bias*_, for the integrated water splitting device under illumination a solar light simulator: (**a**) nanostructured MgB_2_ photoanode and the Pt cathode submerged in deionised water; (**b**) nanostructured MgB_2_ photoanode and the Pt cathode submerged in 0.5 M LiOH electrolyte; (**c**) nanostructured MgB_2_ photoanode and the Pt cathode submerged in 1 M NaOH electrolyte; (**d**) nanostructured MgB_2_ photoanode and the Pt cathode submerged in 0.5 M NaCl electrolyte – sea water; (**e**,** f**) photocurrents generated by blue (*λ* ≈ 440 nm), green (*λ* ≈ 540 nm), yellow-red (*λ* ≈ 600 nm) and red (*λ* ≈ 720 nm) monochromatic light in 0.5 M LiOH and 0.5 M NaCl electrolytes, respectively.

We have tested the efficiency of a MgB_2_ nanostructured photoanode in a two-electrode photo-electrochemical cell configuration with a Pt microwire serving as a cathode (Fig. 1a). The white light was provided by a solar simulator AM 1.5G (Newport) which has illumination intensity ∼100 mW cm^− 2^. The current-voltage (IV) characteristics were recorded using a digital sourcemeter (Keithley, Model 2400) with and without an external potential bias (*V*_*bias*_) applied across the cell. The most efficient MgB_2_ photoanodes have generated photocurrents up to 0.95 mA under *V*_*bias*_ =0.7 V in distilled (DI) water (Fig. 2a, size of MgB_2_ photoanode was ∼1 cm^2^ and size of a Pt cathode was ~ 0.15 cm^2^ for all measurements with two electrodes separated by ∼3 mm). The measurements were also carried out in 1 M NaOH aqueous solution at pH ∼13.6 and in 0.5 M LiOH and 0.5 M NaCl (pH ∼7–8). In the presence of electrolytes, the photocurrents were significantly enhanced for all the investigated MgB_2_-based photoanodes (Fig. 2b-d) as compared to the values plotted in Fig. 2a for the case of DI water. We found that our inexpensive MgB_2_ nanostructures submerged into a 0.5 M LiOH –DI water solution under a small bias (0.4 V) promote the strong enhancement of light-to-photocurrent conversion.

PEC performance was also measured in a 0.5 M NaCl electrolyte emulating seawater under maximal applied *V*_*bias*_=0.4 V. The difference between photocurrent in “on” and “off” state reached its highest value of ∼1.2 mA. Note that we cannot apply *V*_*bias*_ larger than 0.45 V in the cases of using electrolytes because of appearance the periodical jumps of current to very high values > 10 mA which is analogical to ones described in works^38,39^. Observed fluctuations and a slight drop of the measured currents on longer timescales under illumination are mainly caused by the bubble formation on the surface of the MgB_2_ photoanode (which generated O_2_) and the Pt (which generated H_2_). The bubbles can be seen by naked eyes in a dark room under solar illumination (Figure S1, Supplementary Information (SI)). Optical images taken during the formation and growth of bubbles on the water splitting photoanode and cathode were used for the measurement of the gas-evolving reaction rate. The conversion efficiency has been calculated using bubble microscopy^40^. These measurements confirmed 0.95% Faraday efficiency (each two electrons generated ∼0.95 molecules H_2_^33^). Note that the decreasing of currents on longer timescales can be also caused by heating of solution during solar illumination. The dependences of current versus time are restored if change the electrolyte on the fresh (in this case the bubbles are removed and solution is at room temperature) and the measurements are repeated. Therefore, we found that MgB_2_ nanostructures can effectively absorb solar energy facilitating the reaction of water/seawater splitting and deliver the electrons and holes required for generation of hydrogen and oxygen gases. In order to find the optimal value of bias voltage needed to be applied, we used cycling voltammetry (CV). To this end, we cycle the bias voltage applied to the investigated MgB_2_ anodes in the seawater (0.5 M NaCl electrolyte) linearly from the lowest potential − 0.7 V to the highest + 0.7 V and then in reverse, see Figure S2. In the case of a 2-electrode system (anode and cathode), the voltage in the *x*-axes corresponds to the bias applied to the whole device. We have chosen the bias voltage which corresponded to the point where the peak current was observed which provided the most effective value for the maximal efficiency of the photocatalytic process studied.

The enhancement of light-to-photocurrent conversion can be described by incident photon to current conversion efficiency (IPCE) calculated as^1,6^:

$$\:\text{I}\text{P}\text{C}\text{E}=\frac{{\text{J}}_{\text{S}\text{C}}\left(\text{m}\text{A}\:{\text{c}\text{m}}^{-2}\right)\times\:(1.23\text{V}-{\text{V}}_{\text{b}\text{i}\text{a}\text{s}})}{{\text{P}}_{\text{l}\text{i}\text{g}\text{h}\text{t}}\left(\text{m}\text{W}\:{\text{c}\text{m}}^{-2}\right)},$$

where *J*_*SC*_ is short-circuit photocurrent density, *V*_*bias*_ is the applied potential between photoelectrode and counter electrode, *P*_*ligh*t_ is the total irradiation input, and the redox potential of interest is equal to 1.23 V for water oxidation. The integrated efficiency IPCE together with monochromatic IPCE(*λ*) were calculated from measuring the photocurrent *J*_*SC*_ and are presented in Table S1. It was found that the maximal efficiency could be achieved by selection of optimal ions and their concentrations in the electrolyte that provide the maximal ion mobility. The evaluated efficiencies represent the normalised on the square *A* (cm^2^) of the geometrical area of photo-anode or cathode. The highest efficiency of 1.3% (normalised to the anode area) or 6.5% (normalised to the cathode area) for MgB_2_ nanostructure was obtained in the case of PEC filled with solution of 0.5 M of LiOH in the DI water. In the case of seawater splitting the highest efficiency was ∼1.0% (normalised to the anode area) or ∼5% (normalised to the cathode area) and has been achieved under *V*_*bias*_=0.3 V.

To check that the water-splitting reaction is indeed excited by the VIS and near-IR parts of solar spectrum, we have measured the photo-generated current under monochromatic sources of various wavelengths. Figure 2e, f show examples of photocurrents generated by blue *(λ* ≈ 440 nm), green (*λ* ≈ 540 nm), yellow (*λ* ≈ 600 nm) and red (*λ* ≈ 720 nm) monochromatic light approximately the same density power (∼10 mW/cm^2^). Wavelength selection was done by applying a set of band pass filter (FWHM *Δλ*≈ 20 nm, Thorlabs) using the solar simulator as the light source. The experiments were carried out in 0.5 M LiOH and 0.5 M NaCl aqueous solution at pH ∼7. These measurements give evidence that there is significant spectral increase of the effectiveness of photocurrent generation toward the near-IR part of the spectrum. We observe a maximum approximately single-wavelength power conversion efficiency of ∼2.7% under 10 mW cm^− 2^ of 720 nm monochromatic illumination (Table S1). The evaluated IPCE(*λ*) for 720 nm is approximately 2.25 times as large than that of the for 440 nm (Fig. 2e, f). To explore the feasibility of water splitting by utilizing external electrons from excitation of LSPR in the MgB_2_ nanostructures at the red and near IR wavelengths it was additionally tested the monochromatic photocatalysis for *V*_*bias*_=0.2 V (see supplementary information, Figure S3). Obtained data confirm the tendency of the IPCE action spectra presented in the Fig. 2d.

Examination of short-circuit current *I*_*sc*_ yielded nearly linear dependence on the increasing of the wavelength from blue to the near-IR region, as expected in an ideal photovoltaic device. Note that for both electrolytes (0.5 M of LiOH and NaCl) the measured monochromatic IPCE(*λ*) maxima match with the simulated absorbed fraction within the MgB_2_ nanostructures. The numerical absorbed fraction reaches about 90% for the 150–200 nm MgB_2_ particles and about 70% for the 250 nm particles in the spectral range 500–1000 nm for the same layer thickness of 100 nm (Fig. S6).

**Fig. 3:**
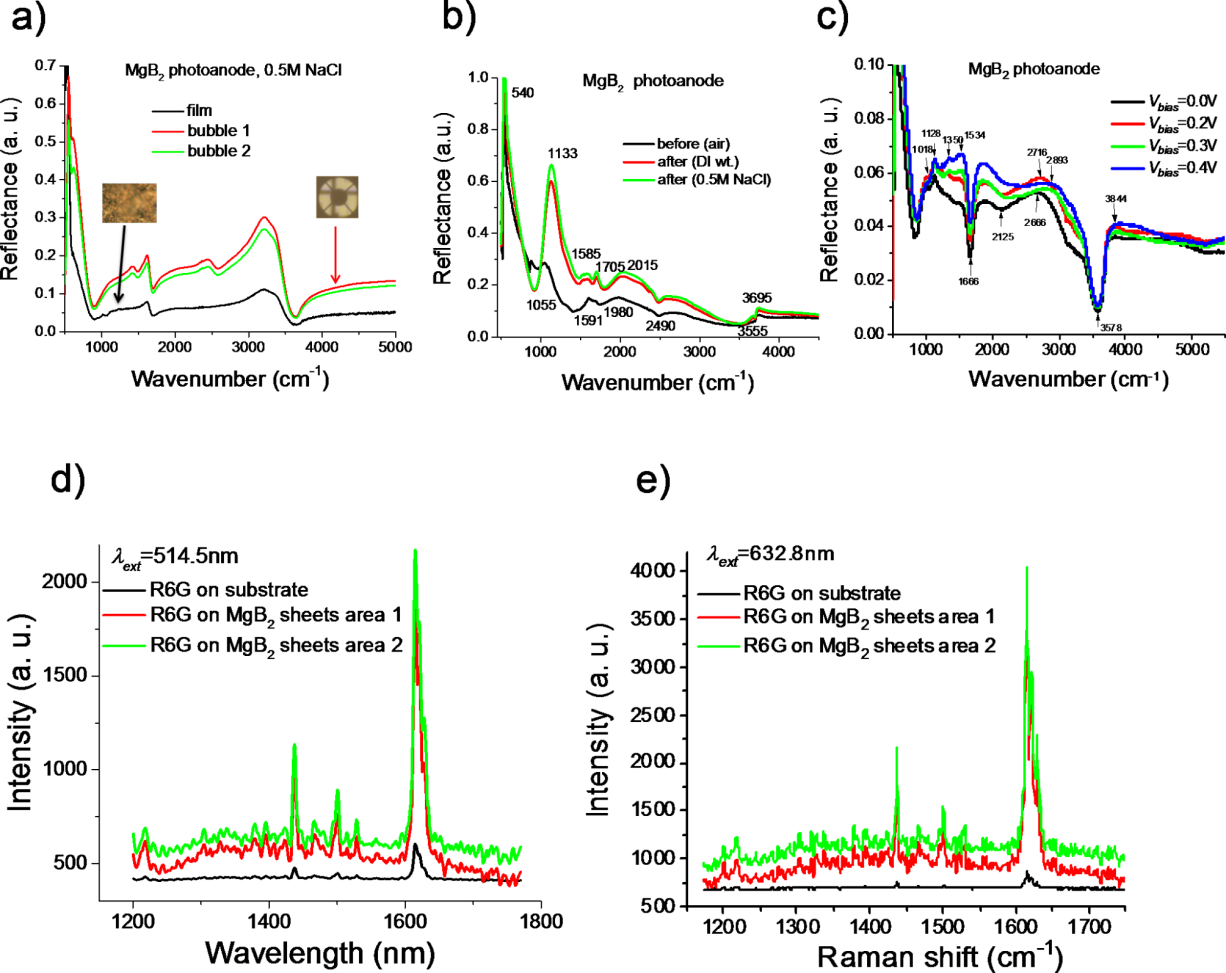
FTIR and Raman spectroscopic results. (**a**) The FTIR spectra in the middle IR region taken from MgB_2_ nanostructure surface in the areas without and with bubbles after water splitting of 0.5 M NaCl electrolyte. Insets (a): Optical images of forming bubbles on the MgB_2_ nanosheets taken by the standard 15x IR (N_A_=0.4) Schwarzschild objective before/after measuring the FTIR spectra. Bubble works as an additional focusing lens. (**b**) FTIR measurements on plasmonic MgB_2_ photo-anode surface before and after water/seawater splitting experiments performed under *V*_*bias*_=0.3 V. (**c**) In situ FTIR spectra of interaction plasmonic MgB_2_ photo-anode with 0.5 M NaCl electrolyte under application of different bias voltage *V*_*bias*_=0¸0.4 V. (**d**, **e**) Surface enhanced Raman signals from R6G dye molecules placed on top of MgB_2_ nanosheet assemblies due to LSPRs of metallic MgB_2_ nanostructures providing highly enhanced electromagnetic fields: (**d**) excitation with *λ* = 514.5 nm and (**e**) excitation with *λ* = 632.8 nm.

**FTIR spectroscopy.** To analyse the contamination of the appeared bubbles on the surface of photoanode (MgB_2_) we employed the Fourier Transform Infra-Red (FTIR) spectroscopy (a Bruker Vertex 80 system with a Hyperion 3000 microscope)^41^. The measurement of the mid-IR reflection spectra was done at normal incidence in the frequency range from 550 to 4000 cm^− 1^ by performing 256 scans with a resolution of 4 cm^− 1^ using cryogenic MCT detector cooled with liquid nitrogen. We carried out FTIR measurements on MgB_2_ nanostructure surface in the areas without and with bubbles in the 0.5 M NaCl electrolyte. The inset of Fig. 3a shows the optical image of forming bubble on the MgB_2_ nanosheets taken by the standard 15x IR (*N*_*A*_=0.4) Schwarzschild objective before/after measuring the FTIR spectra. The spherical surface of bubble performs the additional focusing of incident light and works as lens. As displayed Fig. 3a, new reflection peaks appear for areas contained bubbles. The broad band observed in the range 3000–3500 cm^− 1^ corresponds to the B–OH stretching mode or water bending and indicates possible hydroxyl functionalisation. The peak at ∼628 cm^− 1^ for MgB_2_ nanostructures on bubbles correspond to a B-OH in-plane bending or the B-B in-plane stretching mode and vibration mode at ∼1420 cm^− 1^ associated with B-O stretch. The band at ∼1118 cm^− 1^ can also be attributed to the presence of B–OH functional groups as the B–OH in-plane bending is usually observed to be around 1000–1300 cm^− 1^. The strong band observed at ∼1624 cm^− 1^ can be ascribed to hydrogen motions in B–H–B bridge or water bending. The bubbles of MgB_2_ nanosheets sample also exhibit a moderate band at ∼2445 cm^− 1^, likely due to B–H stretching^36,42^.

Natural seawater is a complex neutral electrolyte, containing main ions such as Cl^−^and Na^+^. The major issues associated with direct seawater photoelectrolysis are: (1) the undesired anodic chloride oxidation reactions - the chlorine evolution (2Cl^−^ → Cl_2_ + 2e^−^) and the formation of hypochlorite (Cl^−^ + 2OH^−^ → ClO^−^ + H_2_O + 2e^−^), competing with the oxygen evolution reaction (OER); (2) the high energy cost caused by the reaction kinetics for hydrogen evolution reaction (HER) and OER in such medium. To examine the selectivity of both oxygen and chloride evolution we employ FTIR for analysis of MgB_2_ photo-anode surface before and after conducting photocurrent measurements (see, Fig. 3b). The fresh MgB_2_ sample displays bands corresponding to OH stretching at 3400–3700 cm^− 1^ and water bending at ∼1650 cm^− 1^, which are indicative of adsorbed moisture. After conducting photocurrent measurements under bias of *V*_*bias*_=0.3 V in water/seawater environments the FTIR spectra of MgB_2_ sample have similar features. The surface of a photoanode exhibits an absorption band at ∼2485 cm^− 1^, likely due to B–H stretching and a band at ∼1585 cm^− 1^ which can be ascribed to hydrogen motions in B–H–B bridge. Strong absorption in the range of ∼1100–1250 cm^− 1^ is characteristic of B–O stretch and hence the band at 1133 cm^− 1^ can be assigned to the presence of oxy-functional groups on boron lattice. FTIR data give evidence that the majority of water molecules at the interface are H- or HO-bounded to the surface.

*In situ FTIR spectra* of an interaction of a photo-anode MgB_2_ with 0.5 M NaCl electrolyte are recorded under applied *V*_*bias*_=0¸0.4 V, see Fig. 3c. They demonstrate an increase of intensity of reflection spectra in the regions of 1000–1500 cm^− 1^ and 2500–3000 cm^− 1^ induced by the applied bias voltage, *V*_*bias*_. The main bands at ∼1665 cm^− 1^ and ∼3577 cm^− 1^ show different bias dependence, see Fig. 3c. The absolute intensity of reflection dip at ∼1665 cm^− 1^ decrease with increasing *V*_*bias*_ while the feature at ∼3577 cm^− 1^ exhibit a much smaller bias dependence. According to Huber^43^, vibration modes associated with Mg–Cl should be expected at < 700 cm^− 1^. They were not observed in the reflection spectra, see Fig. 3b, c. Thus, FTIR study confirms the absence of chlorine production on the surface of MgB_2_ films. It may be possible that a small amount of free chlorine (dissolved Cl_2_ or HClO) could be present but not recorded by FTIR. It is clear that this value is negligible compared to the formed amounts of H_2_ and O_2_.

Chemical surface analysis of the MgB_2_ photo-anodes was carried out using energy dispersive X-ray spectroscopy (EDX) for two samples before (fresh sample) and after (working sample) solar-driven seawater splitting. The SEM and EDX images of the analysed samples and corresponding spectra of the compositions elements are presented in Fig. S4. They show the presence of Mg and B components in the both samples. They also demonstrate the presence of small amount of C, N and O embedded into the MgB_2_ lattice. We further observed an evidence of a small amount of Na on the surface of plasmonic MgB_2_ photoanode after water splitting. An amount of detected Cl was very small indicating the absence of Cl corrosion. The increased amounts of the oxygen on the surface of MgB_2_ photoanodes after conducting photocurrent measurements (right panel in Fig. S4) is, perhaps, due to forming of O_2_ bubbles on MgB_2_ electrode during water splitting. Therefore, results of the EDX measurements provide experimental evidence that supports our optical investigation by FTIR. We are planning to perform gas analysis under seawater splitting in near future.

### Raman spectroscopy

Metallic NPs can contribute to the enhancement of Raman signal through their localized electromagnetic hotspots provided by LSPRs. To test electromagnetic field enhancement due to LSPRs in the fabricated milled MgB_2_ nanostructures, we spin-coated the samples with a 10^− 6^ M of R6G solvent in PMMA 950, 3% anisole and measured Raman spectra of the final system. The Raman measurements were performed using a Witec confocal scanning Raman microscope under a 514.5 and 632.8 nm laser illuminations and a 1800 lines/mm grating, and a ×100 objective (N.A. = 0.90). The laser spot size was approximately ∼3 μm while the laser incident power did not exceed ∼0.2–0.5 mW (to avoid a damage or oxidation of MgB_2_ nanosheets and R6G molecules). The Raman spectra of R6G on pure substrate and substrate-MgB_2_ nanosheets are shown in Fig. 3d, e. The three characteristic peaks at 1437, 1502, and 1613 cm^− 1^ are compared in Fig. 3d for the pure 10^− 6^ M R6G, and combination of MgB_2_ nanostructures and 10^− 6^ M R6G dye layer (about 100 nm of thickness). We revealed that the resonant Raman signal of R6G dye is enhanced by 10–15 times in magnitude by near-fields of plasmonic MgB_2_ nanostructures. Enhancement factors of Raman 1502 and 1613 cm^− 1^ peaks are different for different excitation wavelengths (514.5–632.8 nm). The strongest enhancement in the Raman spectrum was observed under a laser excitation wavelength of 632.8 nm (Fig. 3e), indicating the strong LSPR effect of the laser light scattering on the MgB_2_ nanostructures. Thus, over 15 times enhancement of Raman signal was observed for R6G on nanostructured MgB_2_ substrates. We assume that LSPRs of hybrid MgB_2_ nanosheets concentrate the electromagnetic fields near the surface and edges nanostructures creating plasmonic hotspots on the sharp edges around the perimeter of nanoflakes and in small gaps between neighbouring flakes. These plasmonic hotspots can amplify both the excitation and emission radiation and thus enhance the weak Raman signals from R6G molecules^21,44^.

### Ellipsometric measurement of the complex dielectric function

**Fig. 4:**
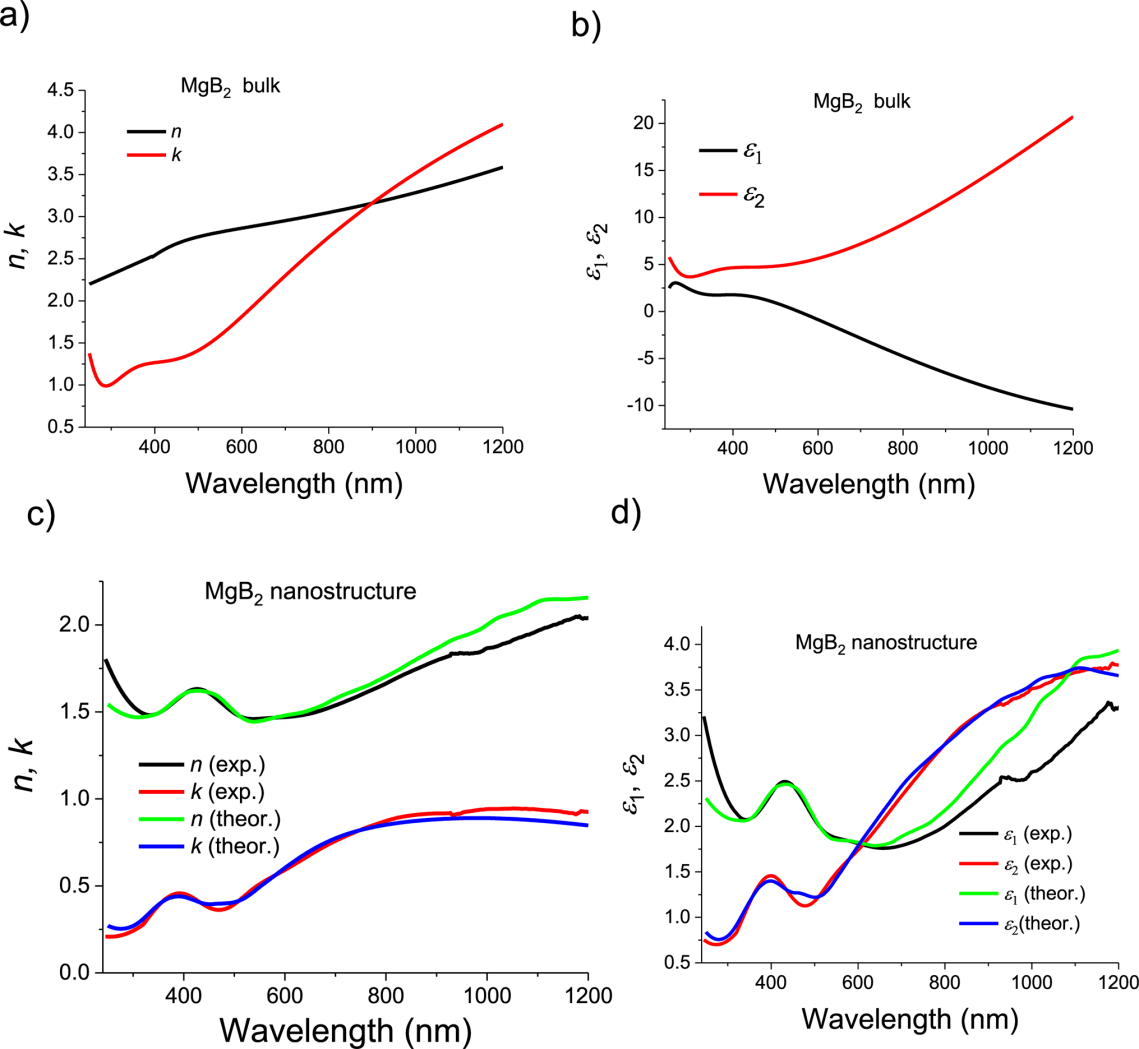
Optical properties of MgB_2_ nanostructured films. (a, b) The real and imaginary parts of the refractive index (**a**) and the dielectric function (**b)** for the plasmonic MgB_2_ bulk metal based on approach of^44^ (Table S2). **(c)** Comparison of the complex refractive index evaluated from ellipsometric measurement and theoretically simulated dependences based on polarizability of the discs-like nanostructures with strong LSPRs. **(d)** The real and imaginary parts of the dielectric function for the plasmonic MgB_2_ nanostructured films: experimental and theoretical dependences.

To determine the complex dielectric function and optical absorption of fabricated MgB_2_ nanostructures, the ellipsometry method was employed using a variable angle focussed-beam spectroscopic ellipsometer Woollam M 2000 F in the wavelength range of 240–1690 nm. The spot size on the sample was approximately 50 μm × 70 μm at ~ 70–80° angles of incidence. The ellipsometry measurement essentially monitors changes in the polarized reflection. It yields two spectral parameters (*Ψ* and *Δ*) related with the amplitude (tan*Ψ*) and phase *Δ* of a complex reflectance ratio *ρ* that provides the ratio of the reflection coefficient for *p*-polarised, *r*_*p*_, and *s*-polarised, *r*_*s*_, light as *ρ* = *r*_p_/*r*_s_ = (tan*Ψ*)exp(*iΔ*)^41,45–47^. The ellipsometric measurements were done at an angle of incidence of light close to *θ*_*i*_ = 73^o^ corresponding to the pseudo-Brewster angle of the sample in VIS. A pseudo-Brewster angle for metal provides a sharp jump in Δ -ellipsometric phase and ellipsometric measurements are the most sensitive and precise near this incident angle^45^. To model the measured ellipsometric data, we applied an isotropic model for thick MgB_2_ layer with the aim to extract values of the complex dielectric function ε*= ε_1_ *+ iε*_2_ or the complex refractive index n*=*n* + *ik*. The fitting of the ellipsometry data were performed using Woollam WVASE32 software in which the complex dielectric function can be extracted using the Fresnel theory. The corresponding real and imaginary parts of the dielectric function ε*= ε_1_ *+ iε*_2_ and refractive index n*=*n* + *ik* are displayed in Fig. 4. Previous analysis of the absorptive part of the dielectric function *ε*_2_ show that main features are associated with the bulk plasmonic peaks in the blue range (~ 400 nm) and broad absorptive region stretched from 500 to 1500 nm. The resonance peaks and dips in the optical spectra in Fig. 4 are the results of specific morphology, i.e. shape, size, and configuration of MgB_2_ nanostructures. Obtained dependences of *ε*_1_ and *ε*_2_ versus wavelength for fabricated MgB_2_ nanostructures are very different from that of bulk MgB_2_ which can be found in the works of Kuzmenko (Fig. 4a-d)^48,49^. It was shown that the plasma edge for bulk MgB_2_ is smeared due to the strong interband transition in the region 400–500 nm (flat shoulders in *ε*_1_ and *ε*_2_ (Fig. 4b)). As a result, the reflectivity spectrum of bulk MgB_2_ in the visible range is relatively flat with a maximum at ∼ 440 nm which demonstrates only blue-silver colour in contrast to multiple colours image of MgB_2_ nanostructures (Fig. 1). It means that different surface electronic states in the bulk and nanostructured MgB_2_ layer lead to different surface collective electronic excitations. We can conclude that the electronic structure of nanoscale MgB_2_ is highly sensitive to LSPRs and uncompensated surface chemical bonds^50^. The experimental results displayed in Fig. 4 also demonstrate that the plasmonic response of MgB_2_ nanostructures can be tuned by adequate choice of the size throughout the visible wavelength range and further into the near-infrared range.

### Plasmonic mechanism of water splitting on MgB_2_ nanostructures

The first direct evidence of excitation of LSPRs in fabricated MgB_2_ nanostructures is multiple colours on the optical microscopy images, Fig. 1c. (The colour of amorphous or crystalline MgB_2_ is normally black.) The second evidence of LSPRs excitation and hence plasmonic mechanism in photocatalytic seawater splitting based on the MgB_2_ nanostructures is the enhancement of Raman light scattering shown in Fig. 3. The third confirmation of the presence of LSPRs in the studied samples lies in the spectral dependence of the real and imaginary parts of the dielectric function for MgB_2_ nanostructures which can be modelled as a sum of excited multi-wavelength LSPRs in the spectral range of 400–1000 nm. Indeed, the dielectric permittivity of bulk MgB_2_ ε*_b_*= ε*_1b_ *+ iε*_2b_ can be described as contributions from the intraband electron transition (the Drude term for electronic response of the free electrons in the conduction band) and interband transitions between two *σ*-bands and from *σ* band to the *π* band close to the M-point of Brillouin zone where a van Hove singularity strongly enhances the density of states^48,49^. The interband electron transitions contribute to the dielectric function as sum of the Lorentz oscillators. The bulk ε*_b_*= ε*_1b_ *+ iε*_2b_ for MgB_2_ can be described using the Drude–Lorentz parameters taken from^48^. The real part of the dielectric function *ε*_1b_ for bulk MgB_2_ becomes negative across a wide range of wavelengths as shown in Fig. 4b. It means that the condition for LSPR excitation (*ε*_1b_≈-2, and a small value of *ε*_2b_) can be established for MgB_2_ metal in air at visible- near IR wavelengths. Thus the combination of *ε*_1b_ ≈ − 2 and low *ε*_2b_ stipulate that nanostructured MgB_2_ could exhibit LSPRs in VIS and near-IR.

To reproduce the complex dielectric function of the fabricated MgB_2_ nanocomposites (Fig. 4) we used the Maxwell-Garnet effective medium approximation (EMA)^46,51^. According to the EMA in the case when incident light wavelength is much larger than the size of the nanostructure, the MgB_2_ nanodiscs-like can be approximated by dipoles with corresponding polarizabilities, *α*_*j*_. The MgB_2_ nanocomposites were considered as discs with a thickness of 25 nm and different diameters in the range from 25 to 500 nm estimated from SEM data (Fig. 1). The volume fraction *f* of the MgB_2_ nanodiscs was found from the best fit as *f* = 0.67 (*f* ≈2/3 of the dimension - volume fraction of MgB_2_ nanodiscs in the air, see in detail SI). Figure 4c, d and supplementary Fig. S5 show the real and imaginary parts of the modelled dielectric function and optical constants of MgB_2_ nanodiscs as a function of wavelength (see SI). Displayed dependence of the dielectric permittivity was obtained as a sum of contribution from assemblies polarised nanodisc-like dipoles with different weighting. As expected from the broad size nanodiscs distribution, we observe a substantial spectral spread in LSPRs leading to an essential broadening of the experimentally measured absorption. The calculated data (Fig. 4c, d) give a reasonable quantitative description for the optical properties of the MgB_2_ nanostructures except in the regions below *λ* < 400 nm and above *λ* > 800 nm where plasmonic resonances of higher orders and scattering on roughness surface should be taken into account. In Figures S6a-c, we show the calculated total absorbed (*A*_*p*_), reflected (*R*_*p*_) and transmitted (*T*_*p*_) fractions of normally incident light in air for MgB_2_ nanodiscs (the calculations were done using approach of our previous works^46,52,53^ (see SI)). Our modelling predicts a sufficient solar light absorption of a nanostructured MgB_2_ photoanode at wavelengths *λ* > 400 nm (Fig. S6) due to a broad size distribution of nanosheets leading to excitation of multi-wavelengths LSPR. Note that most of nanosheets demonstrate an anisotropic shape (SEM images, Fig. 1) which is the reason for plasmon resonances being shifted to the red and near-IR wavelength range.

A plasmonic mechanism of photocatalytic DI and seawater splitting based on the MgB_2_ nanostructures was also confirmed by occurrence of SERS-like signal (Fig. 3d, e) which can be attributed to the electromagnetic origin. In the case of classical plasmonic NPs such as Ag or Au, the process of Raman light scattering dominates due to the plasmon relaxation for relatively large NPs (over ~ 100 nm)^21^, while for smaller (less than ~ 30 nm) and larger (*d* > 300 nm) NPs the e–h pair formation is the dominant process^54^. The excitation of the LSPRs in MgB_2_ nanostructures enhances the weak Raman signals (Fig. 3d, e) in a SERS-like process and significantly enhance the water splitting in photo-electrochemical process (Fig. 2). We also found that plasmonic MgB_2_ photo-anodes display smaller values of the real and imaginary parts of the refractive index, *n + ik*, in comparison to those of thick MgB_2_ (Fig. 4). It is worth noting that *n* is ranged between 1.5 and 1.6 while is 0.5 < *k* < 0.7 (500 < *λ* < 700 nm). Obtained values of the *n* and *k* come close to the perfect absorption conditions in water because the refractive index, *n*, matches condition *n ≈ N*_*wt*_≈1.33 together with a small *k* lead to a small reflection from the interface water-photo-anodes according to the Fresnel theory^55,56^ given by *R*={(*n-N*_*wt*_)^2^ + *k*^2^}/{(*n + N*_*wt*_)^2^ + *k*^2^}≈ *k*^2^*/*(*n + N*_*wt*_)^2^. Moreover, reasonably small imaginary part of the refractive index *k* can affect the strong light absorption in the photo-anode provided 2*πkd/λ*⪢1, where *d≈*50–100 nm is thickness of a photo-anode.

The experimental studies and theoretical modelling demonstrate that Mg- and B- terminated states in the multi-layered MgB_2_ nanosheets are responsible for the excitation of LSPRs and tunable band gaps in the surface states^33,34,50^. Our modelling of *A*_*p*_, *R*_*p*_ and *T*_*p*_ (Fig. S6) showed that assemblies of MgB_2_ nanosheets exhibit evidence of a direct plasmonic absorption, which is distinctly different from previously reported metallic-like interband transitions^48,49^. As nanostructures become more isotropic, i.e., the aspect ratio of diameter to thickness tends toward unity, the LSPRs of the particles become blue-shifted (peak around of 400 nm in the dielectric function, Fig. 4). Note that for a random distribution of nanodiscs with diameters ranging from 100 to 250 nm and a thickness of 25 nm, the plasmonic peaks cover a region from around 600 to 900 nm. The strongest plasmon peak is located at ~ 700 nm since nanodiscs with 200 nm diameter have the highest occurrence in our studied MgB_2_ samples. In addition, smaller particles cause significantly smaller absorption because of their decreased volume. As a result, an assembly of MgB_2_ nanosheets with plasmonic absorption could be better suited to collect VIS and near-IR light for water splitting than wide band gap semiconductors^1,3,13^ and, therefore, can significantly improve the solar-to-energy efficiency of photocatalytic H_2_ generation.

**Fig. 5:**
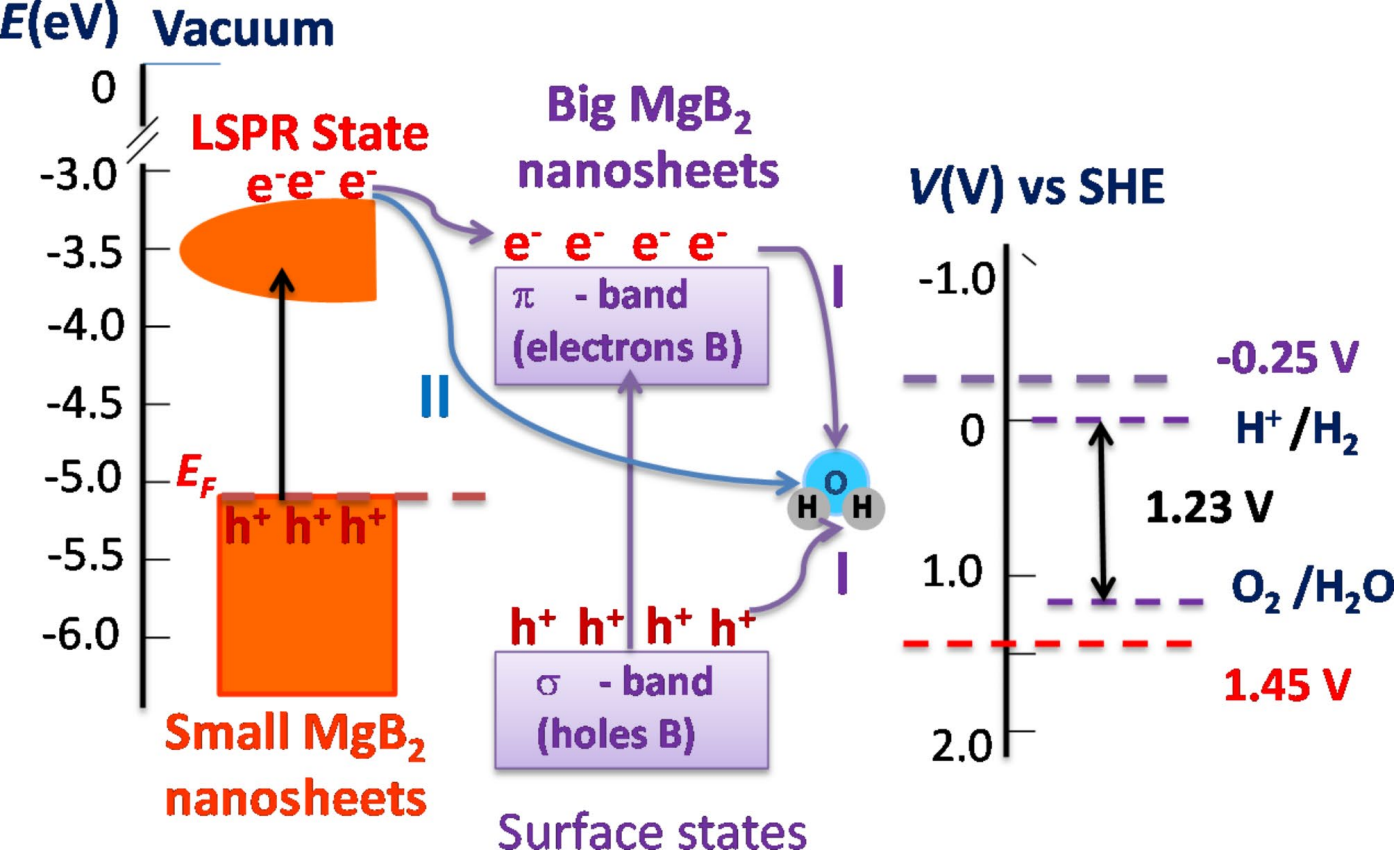
Schematic of plasmon-enhanced water splitting on MgB_2_ nanostructures catalyst. (**a**) Schematic of Fermi-Dirac type distribution of high energy electrons originated from LSPs of small MgB_2_ nanostructures *d* < 300 nm, energy diagram of localisation of the Fermi level (*E*_F_) and LSPRs states for plasmonic MgB_2_ nanosheets and proposed mechanism of hot-electron induced dissociation of H_2_O on MgB_2_ surface (left part of panel). Overview of the energy level positions of surface electrons on the MgB_2_ nanosheets of large sizes *d* > 300 nm with respect to the redox potential of H^+^/H_2_ (0 V vs. SHE). The top potential of surface electrons (*π*-like B) is more negative than the redox potential of H^+^/H_2_ (0 V vs. SHE) while the bottom potential of surface electrons (*σ*-like B) is more positive than the redox potential of O_2_/H_2_O (1.23 V vs. SHE) (right part of panel). Electron-transfer processes locally redistributed electron densities across plasmon/semiconductor-like (indirect transfer (I)) and plasmon/absorbate (H_2_O or electrolyte molecules) interfaces (direct transfer (II)).

A schematic model of a plasmonic photocatalytic mechanism of water splitting with non-noble metals is presented in Fig. 5. As was shown in our previous works^33,34^, electron-hole pairs in MgB_2_ nanostructures are created by interband transitions between *π* and *σ* bands due to the light absorption. This transition originated from a van-Hove singularity in the electronic structure of MgB_2_ and is separated by the energy of ∼ 2.0-2.6 eV^33,57^. Generation of the electron-holes pairs at the metal surface, in analogy with excitons in semiconductors, can occur due to the significant decreasing and slowness of the Debye screening as results of the electron-hole interactions through the surrounding dielectric^54^. Such existence of the electron-holes pairs at the surface is favourable for MgB_2_ because the alternating layers of Mg and B are charged due to ionic/covalent interaction between them^50^. Moreover, metallic Mg layers give electrons to semiconducting B layers and become positively charged while B layers accept electrons and become negatively charged^58^. Electrical stability of the MgB_2_ is provided due to the compensation of the Mg ionic charges by the negative charges on the subsurface B nanosheets. Note that angle-resolved photoemission spectroscopy confirmed the appearance an additional band assigned to a surface state of the terminated Mg- and B-surfaces^59^. From point of view of the electronic structure, we can consider the MgB_2_ nanostructured surfaces as an *n*-type doping of the clean B-terminated surface because the Mg atoms donate electrons interstitially. In the suggested model, we prescribe the semiconductor-like electronic behaviours to the surface of big nanosheets *d* > 300 nm. In order to facilitate the water splitting with semiconductor catalysts, the bottom level of the semiconductor conduction band should be more negative than the redox potential of H^+^/H_2_ (*V*_*H+/H2*_= -4.44 eV vs. vacuum level, or 0 eV relative to the standard hydrogen electrode (SHE)) while the top level of the valence band should be more positive than the redox potential of O_2_/H_2_O (*V*_*O2/H2O*_=- 5.67 eV vs. vacuum level, or 1.23 eV relative to SHE)^4,6^. For MgB_2_, it was found^33,50^ that the B-terminated surface gives a work function of *Φ*_*B*_ ≈ 5.95 eV (∼1.45 eV vs. SHE), with *Φ*_*H*_ ≈ 4.5 eV), while the Mg-terminated surface gives *Φ*_*Mg*_ ≈ 4.25 eV (− 0.25 eV vs. SHE). Energy diagram Fig. 5 shows that the difference between energies, *Φ*_*B*_-*Φ*_*Mg*_, exceed the Gibbs free of water splitting meaning this energy is enough to split one of water molecule into H_2_ and O_2_^4,6^. MgB_2_ nanostructures absorb solar photons and produce electron-hole pairs leading to redistribution of surface electrons and corresponding potentials. Due to a larger number of excited electrons, the top *π*-band becomes more negative than the redox potential of H^+^/H_2_ (0 V vs. SHE) while, due to collection of holes, the bottom *σ*-band of surface electrons tends to obtain a positive potential that is larger than the redox potential of O_2_/H_2_O (1.23 V vs. SHE) (see Fig. 5). It means that energy separation between the *π*- and *σ*-bands would be enough for photocatalytic water splitting reaction^31,34,60^.

Schematic model (Fig. 5) also shows plasmon-induced photocatalytic reactions under LSPR excitations and a LSPR-mediated electron transfer process. Furthermore, the broad distribution of the resonance wavelengths provided by different sizes and shapes of MgB_2_ nanostructures suggests that the entire solar spectrum could be exploited in plasmonic-metal photocatalytic reactions. We can assume that the fabricated plasmonic nanostructures behave as assemblies of electromagnetic dipoles strongly interacting with solar radiation. When a plasmonic nanostructure interacts with light of the same frequency as its own LSPR, the resonance is established between the electronic plasma and the incoming photons. As a result, the electric field in the surroundings of the nanostructure is significantly enhanced. This can influence electron transitions from the *σ* - and *π*-bands in the neighbouring nanostructures and provoke electron–hole pair formation in the phenomenon known as plasmon resonance energy transfer (PRET)^15,20,21^. The plasmon resonance frequency of our photo-anode nanostructured material lies at around 600–800 nm as evident the monochromatic photocatalytic reactions (Fig. 2e, f); thus, we can expect a maximum energy of ∼1.5-2 eV for hot-electrons generated from the LSPRs. Such energetic electrons from plasmonic MgB_2_ nanostructures can be injected into the *π*-band of larger MgB_2_ nanosheets (Fig. 5). From a quantum perspective, the LSPR excited state can be described as a coherent superposition of low-energy electrons and holes near the Fermi level (*E*_F_)^16–18,61^.

Overall, one could assign plasmon-induced water splitting with MgB_2_ nanostructures to a direct plasmon-induced charge transfer conditioned by a significant energy and spatial overlap between the oscillating electron density within MgB_2_ nanosheets and electron-accepting orbitals of H_2_O. Recently, Meng and co-workers theoretically described mechanism of plasmon-induced water splitting at atomic scale and identified the unexpected mode-selectivity in water splitting rate on small metal NPs^62^. They revealed that direct plasmon-induced charge transfer occurs when high electron energy overlap with water’s antibonding state. Being in an exited “hot” state, electrons have enough energy to overcome the energy barrier required for driving the hydrogen evolution reaction (HER) with a redox potential that lies at an excitation energy of about 1 eV above the Fermi energy of the MgB_2_ nanosheets. Such electrons with energies greater than 1 eV (in addition to over-potential) can transfer directly to the unoccupied states of the H_2_O and thus result in the initiation of the photocatalytic HER. Cycling voltammetry scans (Fig. S2) show that the peak response at ∼0.25 V provides еру total energy of electrons as ∼1.25 eV which exceeds the required energy for direct driving of HER. Note that the density of electronic states at the Fermi level is significantly larger for low dimension MgB_2_ nanosheets than that in the bulk MgB_2_^58^ leading to an enhancement of photocatalytic HER due to increased numbers of energetic electrons. Therefore, we can assume that photo-catalytic activities of the studied MgB_2_ nanostructures come from both semiconducting-like MgB_2_ surface states and surface LSPRs arising near terminated Mg- and B- edges (Fig. 5).

In conclusion, a new method of fabrication of larger area flexible plasmonic nanostructure photocatalysts for water and seawater splitting has been suggested. The method is based on a mechanical rolling mill procedure and can be easily implemented by industries. Our study demonstrates effective plasmon-induced seawater splitting with the help of the fabricated MgB_2_ nanostructures. These plasmonic nanostructures consist only of metallic MgB_2_ nanosheets and promise much better efficiency than that of semiconductor photocatalysts often restricted by their large bandgap. The complex dielectric function of the MgB_2_ nanostructures has been determined and confirmed the presence of multi-wavelength LSPRs in the system. Experimental data on monochromatic water splitting and Raman scattering enhancement strongly suggest that the efficiency of photocatalysts depends not only on optical absorption of MgB_2_ nanostructures, but also on their localised plasmons. An excitation of LSPRs in MgB_2_ water splitting nanostructures can offer a series of unique properties and functionalities, including spectral tuneability that can be achieved by varying the nanostructure size and shape, size selectivity, electric field enhancement enabling a boost of photocatalytic hydrogen generation from seawater. We showed that non-noble metal plasmonic nanostructures could allow one to overcome limiting factors of photocatalytic efficiency existing for broad bandgap semiconductors by using the whole range of the solar spectrum that can drive the photocatalytic reactions. We hope that this study will provide a unique approach for guiding further developments of plasmonic photocatalysts.

## Electronic supplementary material

Below is the link to the electronic supplementary material.

Supplementary Material 1

## Data Availability

All data generated or analysed during this study are included in this published article and its supplementary information files.
