# Publisher Correction: Water and seawater splitting with MgB_2_ plasmonic metal-based photocatalyst

**DOI:** 10.1038/s41598-025-90715-8

**Published:** 2025-02-19

**Authors:** Vasyl G. Kravets, Alexander N. Grigorenko

**Affiliations:** Department of Physics and Astronomy, the University of Manchester, Manchester, M13 9PL UK

Correction to: *Scientific Reports* 10.1038/s41598-024-82494-5, published online 07 January 2025

The original version of this Article omitted the Abstract.

The Abstract now reads:

“Plasmonic nanostructures can help to drive chemical photocatalytic reactions powered by sunlight. These reactions involve excitation of plasmon resonances and subsequent charge transfer to molecular orbitals under study. Here we engineered photoactive plasmonic nanostructures with enhanced photocatalytic performance using non-noble metallic MgB2 high-temperature superconductor which represents a new family of photocatalysts. Ellipsometric study of fabricated MgB2 nanostructures demonstrates that this covalent binary metal with layered graphite-like structure could effectively absorb visible and infrared light by excitation of multi-wavelengths surface plasmon resonances. We show that a MgB2 plasmonic metal-based photocatalyst exhibit fundamentally different behaviour compared to that of a semiconductor photocatalyst and provides several advantages in photovoltaics applications. Excitation of localised surface plasmon resonances in MgB2 nanostructures allows one to overcome the limiting factors of photocatalytic efficiency observed in semiconductors with a wide energy bandgap due to the usage of a broader spectrum range of solar radiation for water splitting catalytic reactions conditioned by enhanced local electromagnetic fields of localised plasmons. Excitation of localised surface plasmon resonances induced by absorption of light in MgB2 nanosheets could help to achieve near full-solar spectrum harvesting in this photocatalytic system. We demonstrate a conversion efficiency of ~5% at bias voltage of *Vbias* = 0.3 V for magnesium diboride working as a catalyst for the case of plasmon-photoinduced seawater splitting. Our work could result in inexpensive and stable photocatalysts that can be produced in large quantities using a mechanical rolling mill procedure.”

The original Article has been corrected.

