# Supplementary material for: Water and seawater splitting with MgB2 plasmonic metal-based photocatalyst
Source: Sci Rep. 2025 Jan 7;15:1224. doi: 10.1038/s41598-024-82494-5 (PMC11707196; doi:10.1038/s41598-024-82494-5)
Supplement: Supplementary file 1 — Supplementary Material 1 [file 41598_2024_82494_MOESM1_ESM.docx]

**Supplemantery Information**

**for**

**Water and Seawater Splitting with MgB_2_ Plasmonic Metal-Based Photocatalyst**

Vasyl G. Kravets*, Alexander N. Grigorenko

Department of Physics and Astronomy, the University of Manchester, Manchester, M13 9PL, UK

E-mail: vasyl.kravets@manchester.ac.uk

**Sample fabrication.** The photoanode of the photoelectrochemical (PEC) cell consists from copper foils with thickness of 50-100 μm as a substrate and thin nanostructured MgB_2_. To fabricate MgB_2_ nanostructures we utilised the Pepetools 90 mm Flat Rolling Mill. At the beginning of the fabrication process, we covered the squared surface of a Cu foil of size 20x20 mm^2^ by MgB_2_ diboride powder, weighing approximately 30 mg. This powder was sourced from Aldrich and had a purity of greater than 99%. Then the Cu foil contained MgB_2_ was covered on top with free Cu foils same size. The stack Cu-MgB_2_ (powder)-Cu foils was passed through a rolling mill multiple times, with continuous increasing pressure applied between rolls in order to thin diboride films. This procedure leads to the lateral expansion of the samples by a factor of 1.5-2 times, giving a final size of 30mm x 40mm.

**Photocatalysis study.** The photocatalytic H_2_ production was performed in a 50 ml cylindrical polymer reactor with height 5 cm and diameter 2 cm. The cell schematics are shown in Fig. 1. MgB_2_ nanostructured films on Cu substrates with a size approximately 10 x 10 mm^2^ were submerged in ~20 ml of various solutions: DI water, LiOH, NaOH, NaCl electrolytes (the last one corresponds to seawater). The cathode consisted of a spiral formed Pt cathode with an area of approximately 0.15 cm^2^. Such spiral design allows one to maximise the intensity of the incident light from the solar simulator to pass through and reach the MgB_2_ photoanode. The MgB_2_ photoanode and the Pt cathode were both immersed in an electrolyte solution. Subsequently, the reactor was illuminated by a solar light simulator (Oriel Sol3A, AM 1.5, 400 W Xe, ~100 mW cm^-2^). To separate the two electrodes and don’t block gathering bubbles we used the dielectric rubber ring of 3 mm thickness. The resulting current was collected using a Keithley 2400 sourcemeter and recorded using a LabVIEW programme. The produced photocurrent steeply increased after switching on light until it reached its photochemical equilibrium (plateau on the Fig. 2) and then again steeply decreased in dark condition (light off). The photocurrents were significantly enhanced for the investigated MgB_2_ diboride photoanodes when the applied bias voltage slightly increases from 0.2 to 0.4 V.

Fluctuations of the current with time were caused by bubble formation on the surface of electrodes, which affected the effective surface area of electrodes. The produced H_2_ bubbles (clearly seen by naked eyes, Fig. S1) gathered on the cathode surface of Pt electrode, while O_2_ bubbles were gathered on the MgB_2_ anode. The conversion efficiency has been calculated using bubble microscopy approach [S1-S3] (and following gas chromatography). These measurements confirmed 0.95% Faraday efficiency (each 2 electrons generated ~0.95 molecules H_2_). We want to stress the several indirect observations proving that boiling of water on hot spots is not responsible for the observed bubbles. First, we observe that bubbles are present on both electrodes: bubbles on plasmonic nanostructured MgB_2_ films (which should correspond to O_2_) and bubbles on a flat Pt wires (which should correspond to H_2_). The bubbles on Pt wire cannot be explained by plasmonic hot spots. Second, overall volume of bubbles on the Pt wires was approximately two times larger than the overall volume of bubbles on the MgB_2_ photo-anode which corroborates water-splitting reaction. Third, the overall amount of bubbles on both electrodes strongly increased when an increase of the bias voltage (which would not be the case if the bubbles were produced by water boiling). Fourth, we did perform experiments with heated electrolyte that showed a decrease of photocatalytic activity.

Cycling voltammetry (CV) was used to scan the investigated MgB_2_ photoanodes in the 0.5M NaCl linearly within the bias voltage range -0.7 V to +0.7 V and then in reverse. The measured I-V characteristics are shown in Fig. S2. The bias voltage corresponding to the current peak was chosen to provide maximal efficiency of photocatalytic process.


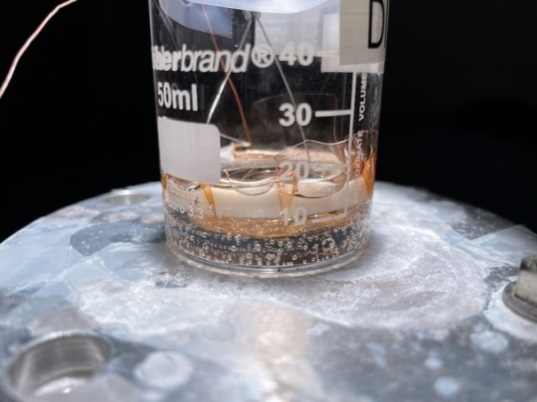


**Figure S1.** Demonstration of bubble formation, perhaps of H_2_ and O_2_, on the surface of electrodes under solar light illumination.

**Figure S2.** Cycling voltammetry scan of the investigated MgB_2_ nanostructured anodes in the 0.5M NaCl (black curves for dark conditions and red ones for the case of illumination).

The incident photon to current conversion efficiency (IPCE) can be calculated as [S4]

$IPCE=\frac{J_{\mathrm{SC}}(mA \mathrm{cm}^{-2})\times(1.23V-V_{\mathrm{bias}})}{P_{\mathrm{light}}(mW \mathrm{cm}^{-2})}$ (1)

where *J_SC_* is short-circuit current density, *V_bias_* is the applied potential between photoelectrode and counter electrode, *P_ligh_*_t_ is the total irradiation input, and the redox potential of interest is equal to 1.23 V for water oxidation. In additional to the conversion efficiencies characterised by the entire solar spectrum on the PEC it is important to understand the spectral efficiency of the photo-generated current. To check that water-splitting reaction is indeed excited by VS-near-IR range of spectrum we have measured the photo-generated current under quasi-monochromatic sources of various wavelengths. Wavelength selection was done by applying a set of band pass filter (FWHM ≈ 20 nm, Thorlabs) at the light source. A solar simulator (calibrated at 100 mW cm^−2^) was used as the light source. Figure 2e,f shows examples of photocurrents generated by blue (*λ*≈440 nm), green (*λ*≈540 nm), yellow (*λ*≈600 nm) and red (*λ*≈720 nm) monochromatic light approximately of the same density power (∼10 mW/cm^2^). The experiments were carried out in 0.5M LiOH aqueous solution at pH ~7. To explore the feasibility of water splitting by utilizing external electrons from excitation of LSPR in the MgB_2_ nanostructures at the red and near IR wavelengths it was additionally tested the monochromatic photocatalysis for *V_bias_*=0.2 V in 0.5M NaCl (Fig. S3a). Obtained data confirm the tendency of the IPCE action spectra presented in the Fig. 2d. Figure S3b shows an absorption spectrum of a plasmonic MgB_2_ photo-anode together with normalised solar simulator AM 1.5G spectral intensity and the spectral dependence of photon to current conversion efficiency (IPCE). The solar photon flux was collected with a uv-visible-near-infrared Ocean Optics USB4000 spectrometer using an optical fibre coupler. The absorption spectrum, *α* was evaluated from the ellipsometric measurements of optical constants *n*, *k*, using equation *α*=2π*k*/*λ* (where *λ* is the wavelength of the incident light). Plasmonic nature of absorption in MgB_2_ nanostructured film enables greater overlap with the solar spectrum. Moreover, the spectral IPCE dependence for the plasmonic MgB_2_ photo-anode clearly shows the correlation with the absorption spectrum, *α.*

The monochromatic photon to current conversion efficiency (IPCE(λ)) can be estimated as [S4]:

$IPCE(\lambda)=\frac{J_{\mathrm{SC}}(mA \mathrm{cm}^{-2})\times1240}{P_{\lambda}(mW \mathrm{cm}^{-2})\times A\times\lambda(nm)}$ (2)

where *λ* is the single wavelength light source, *J*_SC_ (mA cm^−2^) is the differential (light-dark) measured photocurrent for MgB_2_ nanostructured film, *P_λ_* (mW cm^−2^) is the measured power density of the light source at a specific wavelength, *λ* and *A* (cm^2^) is the geometrical area of the illuminated electrode. The number 1240 carries the matching units. Evaluated data of IPCE and IPCE(λ) are gathered and presented in the **Table S1**. These measurements suggest that there is significant spectral increase of the effectiveness of photocurrent generation toward red and near-IR parts of the solar spectrum which is explained by larger number of the photons which can perform water splitting reaction. Note that for both electrolytes (0.5M of LiOH and 0.5M of NaCl) the measured monochromatic IPCE(*λ*) maxima match with the simulated absorbed fraction within the MgB_2_ nanostructures.

**Characterisation of samples**

**Scanning electron microscope images.** The Ultra Plus Carl ZEISS scanning electron microscope (SEM) was used for high-resolution imaging of nanostructures. Ultra Plus SEM with a technology for charge compensation enables high resolution, stable and noise-free images from nanostructured samples. SEM provides information about the samples’ surface morphology. The unique in-lens SEM detector gives resolution of the order of 1.0 nm at 15 kV (1.6 nm at 1 kV), dependent on the type of samples.

**Raman spectroscopy**. Raman spectra of the MgB_2_ nanostructures covered by rhodamine dye R6G dye were recorded with a Witec confocal Raman spectrometer at the excitation wavelengths of 514 nm and 632.8 nm. For this experiment a 10^-6^ M of R6G was solvent in PMMA 950, 3% anisole and then spin coated with thickness of ~100 nm.

**FTIR spectroscopy.** Fourier Transform Infra-Red (FTIR) spectroscopy was performed with the help of a Bruker Vertex 80 system and a Hyperion 3000 microscope. A variety of sources and detectors, combined with aluminium coated reflective optics enable this system to be used from visible to mid-IR wavelengths.

**Table S1**. Collection of the experimental data of photocatalytic reaction on MgB_2_ plasmonic nanostructures. Photochemical water splitting is composed of oxygen evolution reaction (OER) at the anode (MgB_2_) and hydrogen evolution reaction (HER) at the cathode (Pt).

| Electro  lyte | Cathode HER Reaction | Anode  OER Reaction | Current density to anode active area, mA/cm^2^ | Current density to cathode active area, mA/cm^2^ | Efficiency (IPCE) –Anode(Cathode), % | Monochromatic  efficiency (IPCE(λ)), %,  under 10mW/cm^2^ |
| --- | --- | --- | --- | --- | --- | --- |
| H_2_O | 2H^+^ +2e^-^  → H_2_ | 4OH^-^→ O_2_+2H_2_O+4e^-^ | 0.95 (0.7V);  0.7(0.5V) | 4.8 (0.7V);  3.5(0.5V) | 0.7% (3.5%);  0.5% (2.3%) |  |
| LiOH | 4Li^+^+4e^-^+4H_2_O →4LiOH+2H_2_ | 4LiOH→4Li^+^+4e^-^+2H_2_O+O_2_ | 1.25(0.4V);  1.0(0.3V) | 6.25(0.4V)  5.0(0.3V) | 1.3% (6.5%);  1.15% (5.5%) | 2.75%(720nm);  2.2%(600nm);  1.4%(540nm);  1.2%(440nm) |
| NaOH | 4Na^+^+4e^-^+4H_2_O → 4NaOH+2H_2_ | 4NaOH→4Na^+^+4e^-^+2H_2_O+O_2_ | 1.2(0.4V);  0.95(0.3V) | 6.0(0.4V);  4.8(0.3V) | 1.15% (5.6%);  0.95% (4.8%) |  |
| NaCl | 4e^-^+4H^+^ →2H_2_ |  | 1.2(0.4V);  1.1(0.3V) | 6.0(0.4V);  5.5(0.3V) | 1.0% (5.0%);  0.9% (4.5%) | 2.4%(720nm);  1.8%(600nm);  1.1%(540nm);  0.85%(440nm) |

a)

b)

**Figure S3**. **(a)** Photocurrents generated due to illumination of the monochromatic solar light with *λ*≈440 nm, *λ*≈540 nm, *λ*≈600 nm and *λ*≈720 nm by a plasmonic MgB_2_ photo-anode in 0.5M NaCl electrolyte under *V_bias_*=0.2 V at the same power *P*=10 mW. **(b)** Spectral efficiency and a normalised absorption spectrum of a plasmonic MgB_2_ photo-anode together with normalised solar simulator AM 1.5G spectral intensity.

**Energy dispersive X-ray spectroscopy**. Chemical surface analysis of the MgB_2_ photo-anodes was carried out using energy dispersive X-ray spectroscopy (EDX) for two samples before (fresh sample) and after (working sample) solar-driven seawater splitting. The SEM and EDX images of the analysed samples and corresponding spectra of the compositions elements are presented in Fig. S4. They show the presence of Mg and B components in the both samples. They also demonstrate the presence of small amount of C, N and O embedded into the MgB_2_ lattice. We further observed an evidence of a small amount of Na on the surface of plasmonic MgB_2_ photoanode after water splitting. An amount of detected Cl was very small indicating the absence of Cl corrosion. An increased amount of the oxygen on the surface of MgB_2_ photoanodes after conducting photocurrent measurements (right panel in Fig. S4) is, perhaps, due to forming of O_2_ bubbles on MgB_2_ electrode during water splitting. Therefore, results of the EDX measurements provide experimental evidence that supports our optical investigation by FTIR.


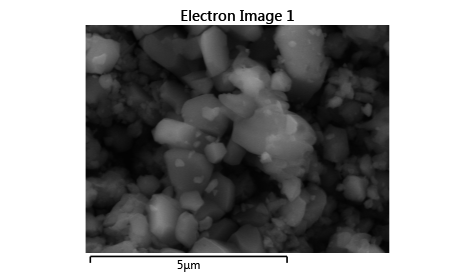

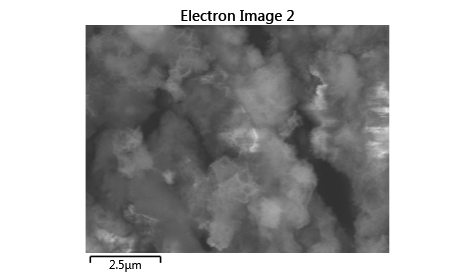


a)

b)


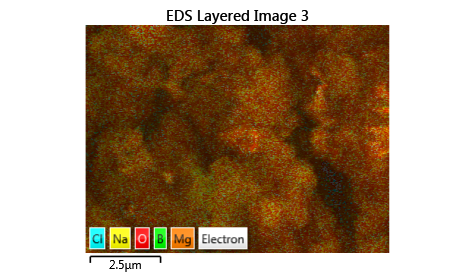

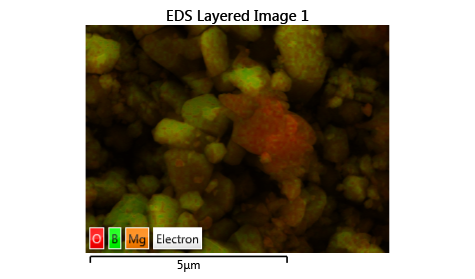


c)

d)


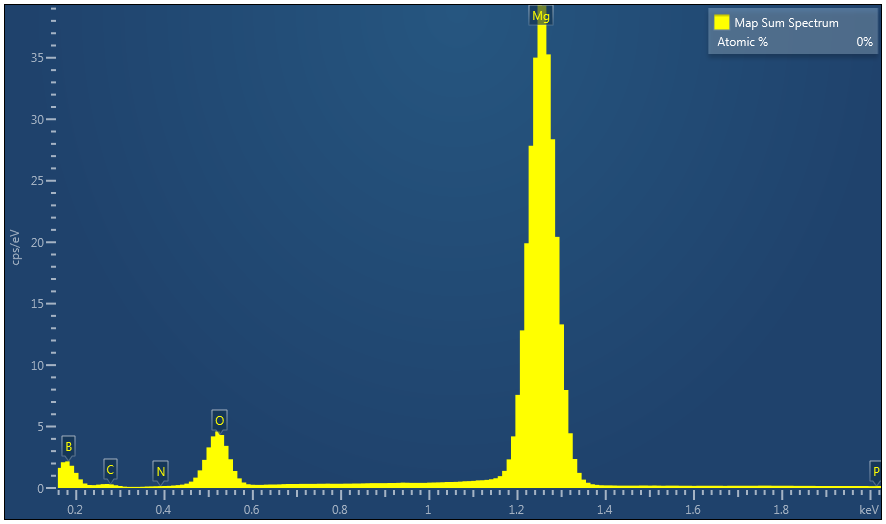

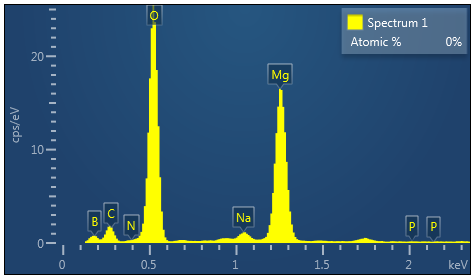


e)

f)

**Figure S4.** Energy dispersive X-ray (EDX) analysis of a plasmonic MgB_2_ photo-anode. The left panels show the images and the EDX spectrum before photo-electrochemical water splitting reaction. The right panels show the images and the EDX spectrum after photo-electrochemical seawater (0.5M NaCl) splitting reaction under *V_bias_*=0.3 V. **(a, b)** SEM image of the analysed area; **(c, d)** EDX image of element distribution in the analysed area; **(e, f)** EDX spectra of the main elements versus X-ray energy.

**Ellipsometric measurements.** To determine the optical absorption of fabricated diborides nanostructures we use the ellipsometry method. We have measured optical constants *n**=*n+*i*k* of the fabricated films on the Cu substrate by employing a variable angle focussed-beam spectroscopic ellipsometer Woollam M 2000F from J.A. Woollam Company (USA). This ellipsometer is based on the rotating polariser-compensator-analyser setup and utilises diode array spectrophotometer to extract spectral ellipsometric parameters. The ellipsometer is equipped with the 75 W Xe arc lamp and measures ellipsometric parameters Ψ and ∆ in the wavelength range of 240-1690 nm. The spot size on the sample was approximately 30 μm × 70 μm for ~60-75° angles of incidence. Ellipsometry measures two parameters: Ψ (ellipsometric reflection) and Δ (ellipsometric phase). These parameters are related to the sample reflection as $\tan(\Psi)\exp\left( i\Delta\right)={r_{p}}/{r_{s}}$, where *r_p_* and *r_s_* are the amplitude reflection coefficients for *p*- and *s*-polarized light. In addition to ellipsometric parameters Ψ and Δ, the ellipsometer allowed us to separately measure *R*_p_=|*r_p_*|^2^ and *R*_s_=|*r_s_*|^2^ providing the intensity reflections for *p-* and *s*-polarised light, respectively. The main ellipsometric formula can be written as [S5] :

$\rho=\frac{r_{p}}{r_{s}}=\tan\psi e^{i\Delta}$ (3)

Using the standard Woollam’s ellipsometric software in the model of bulk combination of assembly diborides nanosheets (Figs. 4, S5) it is possible find the complex optical constants *n**=*n*+i*k* [S5] :

(4)

where measurements were done at *θ_i_*=73^o^ which is the angle of incidence of light and close to the pseudo-Brewster angle in visible region. For this incident angle the ellipsometric measurement is the most sensitive and precise [S5]. From extracted values of the optical constants *n*, *k*, we can estimate the absorption coefficient, *α*=4π*k*/*λ* (where *λ* is the wavelength of the incident light). This absorption coefficient, *α* is the main characteristic of the Lambert’s law: *I*=*I*_0_exp(-*αt*) (where *I*_0_, *I* is the intensity of the incident and transmitted light, respectively, and t is the thickness of film.

**Determining the complex dielectric function of thick MgB_2_ (bulk-like*).*** The optical properties of bulk conductive materials can be generally described by the Drude–Lorentz model for the complex dielectric function [S6]. Drude-Lorentz parameters for fitting the complex dielectric function, *ε*(*ω*)=(*n+ik*)^2^, of thick MgB_2_ were taken from [S7, S8]:

$\varepsilon\left( \omega\right)=\varepsilon_{\infty}-\sum_{i=1}^{2} \frac{\omega_{pi}^{2}}{\left( \omega\left( \omega+i\gamma_{0i} \right) \right)}+\sum_{j=1}^{4} \frac{\Omega_{pj}^{2}}{(\omega_{0j}^{2}-\omega^{2}-i\gamma_{j}\omega)}$ (5)

where, *ε*_∞_ is the high-frequency dielectric constant, *ω_p_* and *γ*_0_ are the plasma frequency and the scattering rate for Drude term of dielectric function, *Ω_p_*^2^, *ω*_0_, *γ* are the oscillator strength, the spectral resonance energy, and the spectral width *γ* of the *j*-th Lorentz oscillator, respectively. The fitting Drude-Lorentz parameters for a thick MgB_2_ are presented in the **Table S2** and the corresponding dielectric function, *ε*(*ω*)=(*n+ik*)^2^  are displayed in Fig. 4. Note that a large scattering rate *γ*_0_=1.1 eV associates with moving of electrons in the *π*-band while a small *γ*_0_=0.03 eV rate can be connected with the *σ*-band electrons [S7, S8].

**Table S2.** The Drude-Lorentz parameters (in eV) for fitting the spectral dependence of the complex dielectric function, *ε*(*ω*)=(*n+ik*)^2^, of thick MgB_2_ (*ε*_∞_=2.9) [S7].

| No. | *ω_p_* | *γ*_0_ | *Ω_p_* | *ω*_0_ | *γ* |
| --- | --- | --- | --- | --- | --- |
| 1 | 1.39 | 0.03 | 1.46 | 0.29 | 0.34 |
| 2 | 4.94 | 1.16 | 3.35 | 0.79 | 1.21 |
| 3 |  |  | 4.85 | 3.40 | 2.40 |
| 4 |  |  | 5.80 | 5.23 | 1.23 |

From plasmonic point of view the nanostructured MgB_2_ behaves as assemblies of electromagnetic dipoles that can interact with external radiation with a large scattering and absorption cross-section. The origin of LSP resonances in conducting nanostructures can be derived for a metal nanodisc that is much smaller than the illumination wavelength. In this case, the disc polarizability can be calculated from the following equation [S9]:

$\alpha\left( \omega\right)=\frac{1}{4}\pi d^{2}t\frac{\varepsilon\left( \omega\right)-\varepsilon_{m}}{\varepsilon_{m}+L(\epsilon\left( \omega\right)-\varepsilon_{m})}$ , (6)

### where *ε*(*ω*) is the dielectric function of the metallic MgB_2_ (Eq. 5), *ε_m_* is the surrounding medium permittivity, which is assumed to be 1 for air, and *L* is the depolarisation factor for disc [S9] and *d, t* is the diameter and thickness of the disc, respectively. We suggest describing the optical properties of fabricated by a rolling milling MgB_2_ nanostructures using the Maxwell–Garnett effective medium approximation (EMA) [S10]. This approach effectively works when the incident light wavelength is much larger than the size of the nanoparticle, and when the nanostructures can be approximated by dipoles with polarizability as shown in (Eq. 6). Within this theory the effective dielectric of the nanostructured MgB_2_ layer can be written as a function of the polarizability of an individual disc, *α*(*ω*) and the depolarisation factor *L* (describing dipole interaction between discs):

### $\boldsymbol{\varepsilon}_{\boldsymbol{eff}}\left( \boldsymbol{\omega} \right)\boldsymbol{=1+}\frac{\boldsymbol{f\alpha(\omega)}}{\boldsymbol{1-fL\alpha(\omega)}}$ , (7)

### where *f* is the volume fraction of the occupied by discs (in our case we set *f*=0.67).

The calculated complex refractive index and dielectric function of MgB_2_ nanostructured film in frame of EMA approach is shown in Fig. 4c,d and S5. We plotted the resulting optical functions originated from assemblies of MgB_2_ nanodiscs with the diameter in range of 25-250 nm. We compare the values of *ε_eff_^*^*(*ω*)= *ε*_1_*_eff_*(*ω*)+i*ε*_2_*_eff_*(*ω*)= (*n_eff_+ik_eff_*)^2^ with the experimental dependences. Ellipsometrically extracted values of the dielectric function *ε_eff_^*^*(*ω*) are in good agreement with values obtained based on EMA modelling. Experimental dependences of the dielectric functions reflected broadband plasmonic excitation from 400 to 900 nm, which was well duplicated by the optical simulation (Fig. S5). Note that the numerical fitting is less accurate for larger wavelengths due to a possible contribution of film roughness and additional impurities to experimental data and in the IR range the Drude term needs to be modified due to the multiple scattering on the grain boundaries. Possible localisation effects also should lead to deviations from plasmonic origin optical response due to weak dielectric-like contributions. The numerical absorbed fraction reaches about 90% for the 150-200 nm MgB_2_ particles and about 70% for the 250 nm particles in the spectral range 400-1000 nm for the same layer thickness of 100 nm (Fig. S6). The evaluated IPCE(*λ*) for 720 nm is approximately two times as large as that of the for 440 nm (Fig. 2 e,f*)*.

We found strong broadband absorption in the visible and near IR region for MgB_2_ nanostructured thin layer. Figures S6a-c show a plot of the transmission, reflection and absorption spectra as a function of the nanodisc diameter, *d*, in the case of the same disc thickness, *t*=25 nm, and thickness of the MgB_2_ layer equal to the 100 nm on glass substrate. These optical spectra are altered by nanoscale changes of the MgB_2_ nanostructures size and shape. With increasing nanodisc size, the magnitude of the plasmon shift in the red region at a given thickness increases (Fig. S6).The transmission of the MgB_2_ nanostructures drops for disc with *d*=150 nm while the absorption is significantly enhanced for this diameter in the spectral range 400-1000 nm. Absorption at wavelengths shorter than 400 nm can mainly be assigned to the interband transition of MgB_2_, which is clearly observed in the absorption spectrum of the small discs, *d*<100 nm (Fig. S6). At wavelengths longer than 550 nm, distinct bands appear for the MgB_2_ with the diameter greater than 100 nm. These results give evidence that MgB_2_ nanostructured thin layer is a suitable absorber for a light-harvesting and may be of an interest for photovoltaic application.

b)

a)

**Figure S5.** Optical properties of MgB_2_ nanostructured films. **(a)** Comparison of the complex refractive index evaluated from ellipsometric measurement and theoretically simulated dependences based on polarizability of the discs-like nanostructures with strong LSPRs. **(b)** The real and imaginary parts of the dielectric function for the plasmonic MgB_2_ nanostructured films: experimental and theoretical dependences.

b)

a)

c)

**Figure S6.** Simulated transmission, reflection and absorption spectra of the MgB_2_ nanostructured layer as a function of the nanodisc diameter, *d*, for disc thickness, *D*=25 nm, and thickness of the MgB_2_ layer equal to the *t*=100 nm on glass substrate. Calculation based on excitation of the LSPRs in the MgB_2_ nanostructures: (a) transmission spectra, (b) reflection spectra, (c) absorption spectra.

**References**

S1. Leenheer, A. J., Atwater, H. A. Water-splitting photoelectrolysis reaction rate via microscopic imaging of evolved oxygen bubbles. *J. Electrochem. Soc.* **157,** B1290–B1294 (2010)

S2. Kravets, V. G., Grigorenko, A. N. A new class of photocatalytic materials and a novel principle for efficient water splitting under infrared and visible light: MgB_2_ as unexpected example. *Opt. Express* **23**, A1651–A1663 (2015).

S3. Kravets, V. G., Thomas, P. A., Grigorenko, A. N. Metallic binary alloyed superconductors for photogenerating current from dissociated water molecules using broad light spectra. *J. Renewable and Sustainable Energy* **9**, 021201 (2017).

S4. Chu, Sh.; Li, W., Yan, Y., Hamann, T., Shih, I., Wang, D., Mi, Z. Roadmap on solar water splitting: current status and future prospects. *Nano Futures* **1**, 022001 (2017).

S5**.** Azzam, R. M. A., Bashara, N.M. *Ellipsometry and Polarized Light.* North-Holland, 1987.

S6. Wooten, F. *Optical Properties of Solids.* Academic Press, New York and London, 1972.

S7. Kuzmenko, A. B., Mena, F. P., Molegraaf, H. J. A., van der Marel, D., Gorshunov, B., Dressel, M., Mazin, I. I., Kortus, J., Dolgov, O. V., Muranaka, T., Akimitsu, J. Manifestation of Multiband Optical Properties of MgB_2_**.**  *Solid State Commun*. **121**, 479-484 (2002).

S8. Guritanu, V., Kuzmenko, A. B., van der Marel, D., Kazakov, S. M., Zhigadlo, N. D., Karpinski, J. Anisotropic Optical Conductivity and Two Colors of MgB_2_. *Phys. Rev. B* **73** (*10*), 104509 (2006).

S9. Bohren, C. F., Huffman, D. R. *Absorption and Scattering of Light by Small Particles*. Wiley, New York, 1983.

### S10. García-Vidal, F. J., Pitarke, J. M., Pendry, J. B. [Effective Medium Theory of the Optical Properties of Aligned Carbon Nanotubes](https://journals.aps.org/prl/abstract/10.1103/PhysRevLett.78.4289). *Phys. Rev. Lett.* 78, 4289-4292 (1997).
